# Evaluation of Recombinant Foot-and-Mouth Disease SAT2 Vaccine Strain in Terms of Antigen Productivity, Virus Inactivation Kinetics, and Immunogenicity in Pigs for Domestic Antigen Bank

**DOI:** 10.3390/vaccines13070704

**Published:** 2025-06-28

**Authors:** Jae Young Kim, Sun Young Park, Gyeongmin Lee, Mijung Kwon, Jong Sook Jin, Jong-Hyeon Park, Young-Joon Ko

**Affiliations:** Center for Foot-and-Mouth Disease Vaccine Research, Animal and Plant Quarantine Agency, 177 Hyeoksin 8-ro, Gimcheon-si 39660, Gyeongsangbuk-do, Republic of Korea; ivorikim@korea.kr (J.Y.K.); sun3730@korea.kr (S.Y.P.); lgm6004@korea.kr (G.L.); alwjd3920@naver.com (M.K.); in75724@naver.com (J.S.J.); parkjhvet@korea.kr (J.-H.P.)

**Keywords:** foot-and-mouth disease, SAT 2, antigen productivity, inactivation kinetics, antigen bank

## Abstract

Background: Since the massive outbreak of foot-and-mouth disease (FMD) in South Korea in 2010–2011, cloven-hoofed livestock have been immunized with serotype O and A vaccines across the country. Other serotypes of FMD vaccines were stockpiled in overseas FMD vaccine factories as antigen banks. Once a manufacturing facility has been established in South Korea, the overseas antigen banks will be replaced by domestic one. Therefore, this study aimed to evaluate the commercial potential of the previously developed SAT2 vaccine candidate (SAT2 ZIM-R). Methods: The optimal condition was determined at various virus concentrations, infection times, and pH levels, resulting in 0.01 MOI for SAT2 ZIM-R for 24 h infection at a pH of 7.5. Results: When the SAT2 ZIM-R virus was produced in flasks from 40 to 1000 mL in fivefold increments, all scales of production yielded > 7.0 µg/mL of antigens. Using a bioreactor, 5.6 µg/mL of antigens was recovered from a 1 L viral culture. The optimal conditions of viral inactivation kinetics were determined to be 1 mM of binary ethyleneimine (BEI) treatment at 26 °C for 24 h, with approximately 91% of the antigen being retained after virus inactivation. When the SAT2 ZIM-R experimental vaccine was administered twice to pigs, the neutralizing antibody titer increased approximately 500-fold after booster immunization. Conclusions: To the best of our knowledge, this is the first study to evaluate the antigen productivity, viral inactivation kinetics, and immunogenicity of the SAT vaccine strain in pigs. In the future, the SAT2 ZIM-R vaccine may be a useful candidate vaccine for a domestic antigen bank.

## 1. Introduction

Foot-and-mouth disease (FMD) is a highly contagious disease that affects ungulates. Although its mortality rate is low, the rapid spread of the disease leads to significant economic losses in the livestock industry. Therefore, FMD is classified by the World Organization for Animal Health (WOAH) as one of the most important transboundary animal diseases, and its occurrence is a major constraint on the international trade of livestock products [1].

FMD virus (FMDV) is a positive-sense single-stranded RNA virus belonging to the genus *Aphthovirus* in the family Picornaviridae [2]. The capsid gene is translated into a polyprotein that is cleaved by viral proteases into structural and nonstructural proteins. Viral particles form an icosahedral protein capsid composed of 60 protomeric units [1,3,4]. The capsid comprises four structural proteins: VP1, VP2, VP3, and VP4. Among these, VP1, VP2, and VP3 are exposed on the surface, whereas VP4 is located internally. During the early phase of translation, structural proteins assemble into protomers consisting of VP0 (a precursor of VP2 and VP4), VP3, and VP1. Five protomers form a 12S pentamer, and 12 pentamers assemble into a 75S empty capsid precursor. Upon RNA encapsidation, VP0 is spontaneously cleaved into VP2 and VP4, thereby forming a mature 146S FMDV particle [5,6,7,8,9]. Although 146S and 12S particles share the same capsid structure, the immunogenicity of 146S particles is approximately 100 times greater than that of 12S particles [10]. Therefore, when measuring the amount of FMD vaccine antigen, it is not a measure of the unit protein content, such as VP1, but of the 146S antigen, which is the entire FMDV particle.

The SAT2 serotype is classified into 14 topotypes (I–XIV) and exhibits greater antigenic variability than the other FMDV serotypes [11]. It remains endemic to Africa and the Middle East [12], and several outbreaks have been reported in regions such as Iraq, indicating its potential for transboundary introduction [10].

South Korea has experienced outbreaks of FMD types O and A and has been vaccinating livestock nationwide with a bivalent FMD vaccine. Vaccines for other serotypes of FMD that have not yet occurred in South Korea, namely Asia1, C, SAT1, SAT2, and SAT3, are currently stockpiled overseas as antigen banks. For domestic production of the FMD vaccine, our organization developed several FMD vaccine candidates, including SAT2, and confirmed their efficacy in pigs [13].

For an in-house vaccine candidate to be used in the field, the antigen must be efficiently produced at an industrial scale, and the virus must be fully inactivated. In this study, we evaluate the commercial potential of the previously developed recombinant SAT2 vaccine strain SAT2 ZIM-R [13]. To confirm its feasibility, we demonstrate high antigen productivity by scaling up production from flask culture to a bioreactor. Additionally, we verify complete viral inactivation via binary ethyleneimine (BEI) treatment. Finally, we confirm that the inactivated antigen produced in the bioreactor retains immunogenicity in pigs.

## 2. Materials and Methods

### 2.1. Cells and Viruses

Suspension-adapted baby hamster kidney (BHK-21) cells were established and cultured in serum-free medium as previously described [14]. Cells were grown in ProVero-1 culture medium (Lonza Ltd., Basel, Switzerland) at 37 °C in a 5% CO_2_ environment at 110 rpm. Porcine kidney (LFBK) cells (Plum Island Animal Disease Center, Orient, NY, USA) and adherent BHK-21 cells (C-13, ATCC CCL-10, Manassas, VA, USA) were maintained in Dulbecco’s modified Eagle’s medium (Thermo Fisher Scientific, Waltham, MA, USA). The SAT2 ZIM-R was constructed via genetical engineering, replacing the structural protein region of the full-length FMDV O1 Manisa/Turkey/69 clone (pO-Manisa-FG) with the SAT2 ZIM 5/81 sequence as previously reported [13].

### 2.2. Determination of Optimal Conditions for SAT2 ZIM-R Antigen Production

BHK-21 suspension cells were cultured in Cellvento BHK-200 medium (Merck, Darmstadt, Germany) for 3.5 days until reaching a density of approximately 3 × 10^6^ cells/mL, starting from 3 × 10^5^ cells/mL in a volume corresponding to 70% of the total volume. The cells were then infected with SAT2 ZIM-R at multiplicities of infection (MOI) of 0.001, 0.005, 0.01, and 0.05 and simultaneously supplemented with 30% (*v*/*v*) fresh Cellvento BHK-200 medium. Viral proliferation proceeded in a shaking incubator at 37 °C with 5% CO_2_. At 12, 16, 20, and 24 h post-infection (hpi), viral suspensions were harvested at different hpi and followed by centrifugation at 4000× *g* for 10 min at 4 °C to remove cellular debris.

### 2.3. Determination of Optimal pH for SAT2 ZIM-R Antigen Production

BHK-21 suspension cells were cultured in Cellvento BHK-200 medium for 3.5 days until attaining 3 × 10^6^ cells/mL, starting from 3 × 10^5^ cells/mL in a volume corresponding to 70% of the total volume. The pH of the culture medium was adjusted to 6.0, 6.5, 7.0, 7.5, 8.0, 8.5, and 9.0 at the time of virus infection. The cells were infected with SAT2 ZIM-R at 0.01 MOI and incubated in a shaking incubator at 37 °C with 5% CO_2_ while simultaneously supplemented with 30% (*v*/*v*) fresh Cellvento BHK-200 medium. Viral suspensions were harvested at 24 hpi, followed by centrifugation at 4000× *g* for 10 min at 4 °C to remove cellular debris.

### 2.4. Virus Titration

Viral titers were determined using the Spearman–Kärber method and adherent BHK-21 cells [15]. Titers are expressed as a 50% tissue culture infectious dose (TCID_50_/mL).

### 2.5. Quantification of FMDV Particles

The amount of FMDV 146S particles was quantified as previously described [16]. Briefly, viral supernatants were mixed with chloroform (Merck) at a 1:1 (*v*/*v*) ratio and vigorously vortexed for 5 min. Then, the solution was centrifuged at 4000× *g* for 15 min at 4 °C, and the aqueous phase was collected. Benzonase (Sigma-Aldrich, St. Louis, MO, USA) was added at a quantity of 0.025 units/µL, and the samples were incubated at 37 °C for 1 h with 100 rpm of agitation. The supernatant was collected after centrifugation at 16,000× *g* for 10 min at 4 °C. FMDV particles were quantified using a high-performance liquid chromatograph (Agilent Technologies, Santa Clara, CA, USA) with a TSKgel G4000PWXL column (TOSOH Bioscience, Tokyo, Japan).

### 2.6. Preparation of Vaccine Antigen Using a Flask Shaker and 2 L Bioreactor

For the flask culture, BHK-21 suspension cells were seeded at 3 × 10^5^ cells/mL at volumes of 28 mL, 140 mL, and 700 mL and cultured for 3.5 days to reach approximately 3 × 10^6^ cells/mL. The cells were then infected with SAT2 ZIM-R at 0.05 MOI using fresh Cellvento BHK-200 medium volumes of 12 mL, 60 mL, and 300 mL, respectively. Virus-infected suspensions were incubated in a shaking incubator at 37 °C with 5% CO_2_ for 24 h.

For bioreactor cultures, BHK-21 cells were seeded at 3 × 10^5^ cells/mL in 700 mL of Cellvento BHK-200 medium and cultured in a 2 L bioreactor (Sartorius, Göttingen, Germany). The bioreactor was equipped with sensors to monitor temperature and dissolved oxygen, maintained at 37 °C with 45% air saturation, respectively. The pH was controlled to 7.2–7.4 using CO_2_ and 0.5 N NaOH. The agitation speed was 150 rpm. Once the cell density reached approximately 3 × 10^6^ cells/mL, 300 mL of fresh medium was added, and the pH was adjusted to 7.5. The suspension was then infected with SAT2 ZIM-R at 0.01 MOI, and supernatants were harvested after 24 h by centrifugation at 4000× *g* for 10 min at 4 °C to remove cellular debris.

### 2.7. FMDV Inactivation Kinetics

A 0.1 M BEI solution was prepared by dissolving bromoethylamine hydrobromide (Sigma-Aldrich) in 10 mL of 0.2 N sodium hydroxide (Sigma-Aldrich), which was incubated in an incubator at 100 rpm and 37 °C for 1 h. It was adjusted to a pH of 8.5–9.0, and the solution was freshly prepared prior to use.

For investigation of the inactivation kinetics, 100 mL of virus-infected supernatant was treated with BEI at different concentrations ranging from 0.5 mM to 3 mM. Samples were incubated at either 26 °C or 37 °C and agitated at 75 rpm for up to 24 h. Aliquots (12 mL) were collected at hourly intervals for the first 6 h and again at 24 h post-treatment.

To neutralize residual BEI, 10% (*v*/*v*) sodium thiosulfate (Daejung Chemicals, Siheung-si, Korea) was added at a final concentration of 2% (*v*/*v*).

### 2.8. Animal Experiments

Purified SAT2 ZIM-R antigens (15 µg/dose) from flask and bioreactor cultures were formulated into a monovalent vaccine by mixing with 1% saponin (Sigma-Aldrich) and 10% aluminum hydroxide gel (General Chemical, Moorestown, NJ, USA). The formulation was emulsified with ISA 206 VG adjuvant (Seppic, Paris, France) at a 1:1 (*v*/*v*) ratio to a final volume of 2 mL/dose.

The emulsion was incubated at 20 °C in a water bath shielded from light for 1 h and stored at 4 °C until use. Five FMD vaccine-naïve pigs (2 months old) received two immunizations with the monovalent vaccine at 4 week intervals. Three unvaccinated pigs served as controls.

Blood samples (10 mL) were collected 0, 7, 14, 21, 28, 35, 42, 49, and 56 days after the initial vaccination. Animal experiments were conducted in accordance with the guidelines and approval of the Animal Ethics Committee of the Animal and Plant Quarantine Agency (APQA; approval number 2024-1158).

### 2.9. Virus Neutralization Test

Virus neutralization (VN) assays were performed according to the WOAH Terrestrial Manual [17]. Serum samples were heat-inactivated at 56 °C for 30 min prior to testing. Twofold serial dilutions starting at 1:4 were prepared in 50 µL volumes and mixed with 50 µL of SAT2 ZIM-R at a concentration of 100 TCID_50_. After 1 h of incubation at 37 °C, 50 µL of LFBK cells (0.5 × 10^6^ cells/mL) was distributed into each well. The plates were sealed and incubated at 37 °C with 5% CO_2_ for 2–3 days. VN titers were calculated as the reciprocal of the highest serum dilution, neutralizing 100 TCID_50_ of SAT2 ZIM-R, and expressed as log10.

### 2.10. ELISA for the Detection of SP Antibodies Against FMDV SAT2

The solid-phase competitive ELISA for antibodies specific to FMDV SAT2 (IZSLER Biotecnology Laboratory, Brescia, Italy) was employed to detect SP antibodies against SAT2 in swine sera. The optical density (OD) values of samples were expressed as the percentage inhibition (PI) relative to the reference OD. Test sera were considered positive when producing an inhibition more than 70%.

### 2.11. Statistical Analysis

All experiments were performed in triplicate, and the results are presented as the means ± standard deviations. Statistical analyses were performed using GraphPad Prism 9 (GraphPad Software, La Jolla, CA, USA). Differences were evaluated using two-way ANOVA, with the significance threshold set at *p* < 0.05.

## 3. Results

### 3.1. Optimization of Conditions for SAT2 ZIM-R Antigen Production

**Figure 1:**
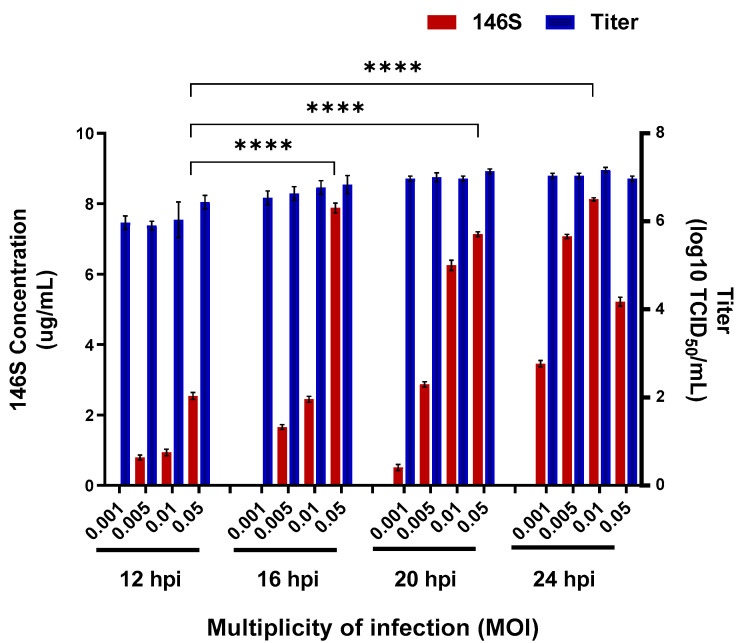
Optimization of antigen production conditions at flask scale. Antigen productivity and viral titer were evaluated based on virus concentration and infection duration. Viral titer is represented by blue bars, and red bars correspond to antigen yield. Data are shown as means ± standard deviations. Statistical comparisons were performed using unpaired Student’s *t*-test (**** *p* < 0.0001).

Virus titers and antigen productivity (146S) were evaluated under various infection times and MOI (Figure 1). Virus titers ranged from 5.9 to 7.2 log10 TCID_50_/mL. The antigen productivity differed significantly, depending on the infection conditions. At MOI values of 0.001, 0.005, and 0.01, the amount of vaccine antigen increased with the viral infection time. However, at 0.05 MOI, the antigen productivity increased up to 16 hpi but decreased at 24 hpi. Therefore, the optimal condition for producing vaccine antigens using SAT2 ZIM-R was determined to be infection of BHK-21 cells at 0.01 MOI for 24 h, yielding an average antigen productivity of 8.1 µg/mL.

### 3.2. Determination of Optimal pH for SAT2 ZIM-R Antigen Production

**Figure 2:**
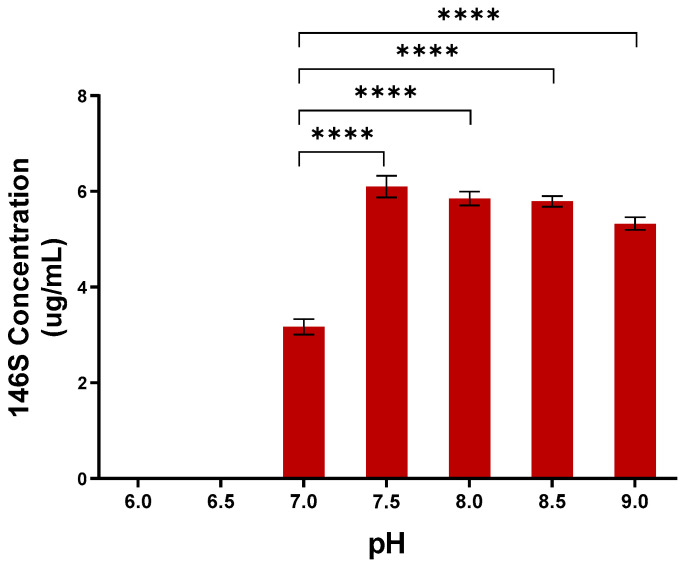
Determination of optimal pH for SAT2 ZIM-R antigen production. Antigen productivity was evaluated based on the medium pH during the virus inoculation phase. Data are shown as means ± standard deviations. Statistical comparisons were performed using unpaired Student’s *t*-test (**** *p* < 0.0001).

To determine the optimal pH for antigen production, the culture medium was adjusted to 6.0–9.0 at the time of SAT2 ZIM-R infection. No antigen was detected at pH levels of 6.0 and 6.5. At pH levels of 7.0, 7.5, 8.0, 8.5, and 9.0, antigen productivity was 3.2 µg/mL, 6.1 µg/mL, 5.9 µg/mL, 5.8 µg/mL, and 5.3 µg/mL, respectively (Figure 2). Thus, all conditions above a pH of 7.5 yielded more than 5.0 µg/mL of antigen, and the pH level of 7.5 was selected as the optimal pH for SAT2 ZIM-R antigen production after viral inoculation.

### 3.3. Comparison of Antigen Productivity According to Production Scale

**Figure 3:**
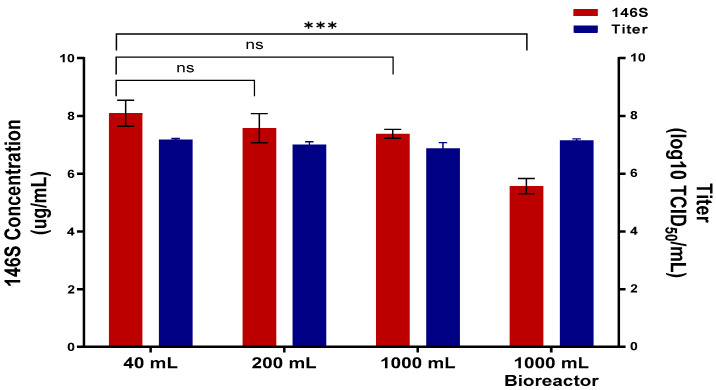
Evaluation of antigen productivity across different production scales. SAT2 ZIM-R antigen was produced by sequentially increasing the culture volume in 5-fold increments with antigen productivity and titer measured at each stage. Viral titer is represented by blue bars, and red bars correspond to antigen productivity. Data are shown as means ± standard deviations. Statistical comparisons were performed using unpaired Student’s *t*-test (ns = not significant; *** *p* < 0.001).

Antigen production was evaluated at different culture volumes under the optimal conditions established above. When the SAT2 ZIM-R virus was cultured in 40 mL, 200 mL, and 1000 mL flasks, antigen productivity was 8.1 µg/mL, 7.6 µg/mL, and 7.4 µg/mL, respectively. The corresponding virus titers ranged from 6.9 to 7.2 log10 TCID_50_/mL (Figure 3). In a 2 L bioreactor using the same optimized conditions, the virus titer was 7.2 log10 TCID_50_/mL, equivalent to that of the flask cultures, and the antigen productivity was 5.6 µg/mL.

### 3.4. BEI Inactivation Kinetics of SAT2 ZIM-R

**Figure 4:**
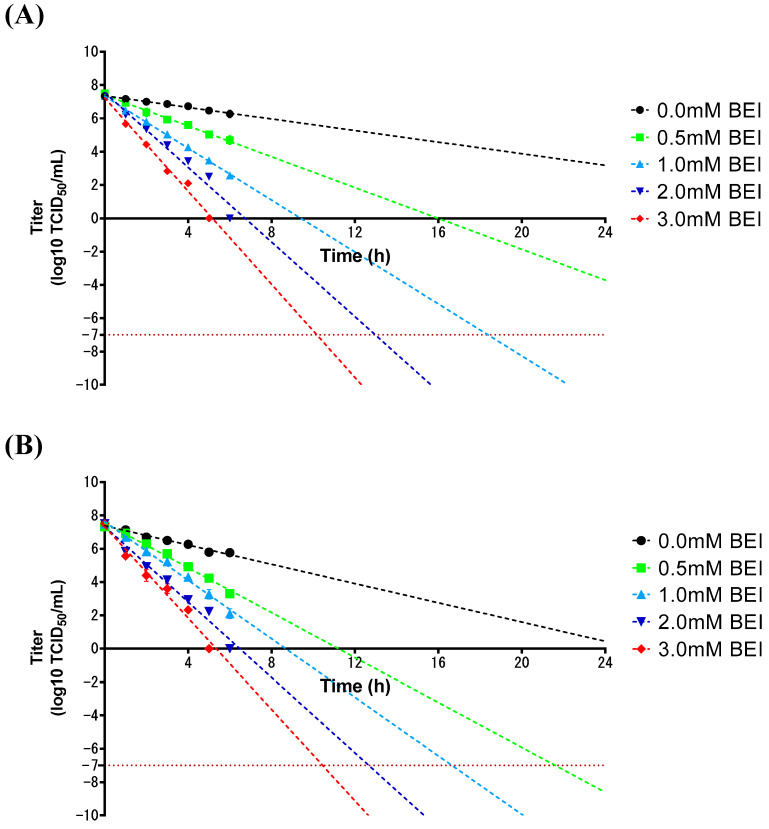
Inactivation kinetics of SAT2 ZIM-R. After inoculating SAT2 ZIM-R into BHK-21 cells in a suspension, the supernatant was treated with various binary ethyleneimine (BEI) concentrations. Samples were collected hourly up to 6 h at 26 °C (**A**) and 37 °C (**B**). Linear extrapolation of individual graphs was performed to analyze the time to decrease viral titers to −7 log10 TCID_50_/mL, which is indicated by a horizontal dotted line.

The inactivation kinetics of the SAT2 ZIM-R virus were assessed using virus-infected supernatants obtained from flask cultures. Viral titers were measured at hourly intervals for the first 6 h following BEI treatment, and linear regression analysis was used to estimate the time required to reduce the titers below −7 log10 TCID_50_/mL. The inactivation rate was faster at 37 °C than at 26 °C, as shown in Figure 4. The optimal inactivation conditions were determined to be 1 mM and 0.5 mM BEI at 26 °C and 37 °C, respectively.

The amount of antigen was measured before inactivation and 6 h and 24 h after inactivation (Table 1). The initial antigen level was 4.19 µg/mL. After treatment with 1 mM BEI at 26 °C, the residual antigen level was 3.8 µg/mL, representing a 9% reduction. Under 0.5 mM BEI at 37 °C, the antigen level decreased to 3.64 µg/mL, corresponding to a 13% reduction. In comparison, BEI-untreated controls exhibited 3% and 10% reductions at 26 °C and 37 °C, respectively. These findings suggest that 1 mM BEI at 26 °C represents an optimal condition for effective viral inactivation while minimizing antigen loss.

### 3.5. Immunogenicity of pSAT2-ZIM-R in Pigs

The SAT2 ZIM-R antigen produced in a bioreactor (15 µg/dose) was administered twice to pigs. Following the first immunization, the ELISA results were generally negative; however, all animals seroconverted after the second immunization.

**Figure 5:**
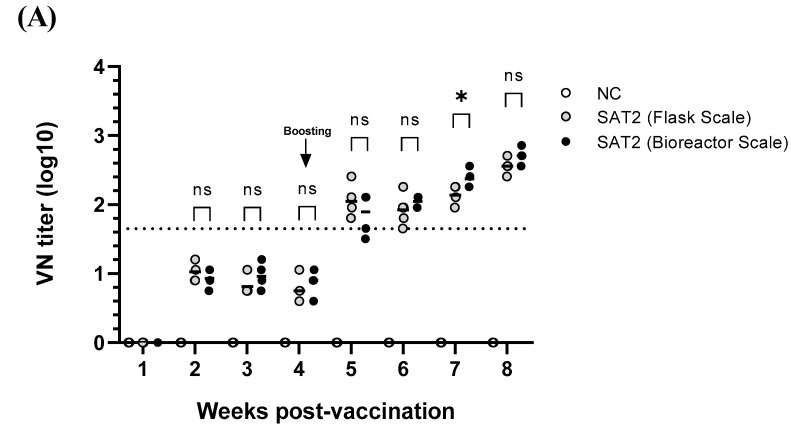

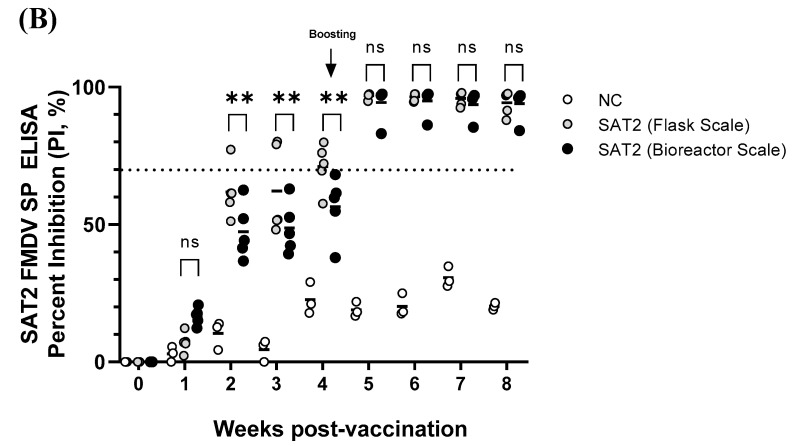
Virus neutralization (VN) titers (**A**) and SAT2 SP ELISA results (percent inhibition (PI%)) (**B**) in pigs following immunization with SAT2 ZIM-R vaccine. VN tests were performed using sera collected weekly from pigs that received two doses of SAT2 ZIM-R vaccine at 4 week intervals. Vaccine antigens were produced using both flask and bioreactor systems. Data are presented as means ± standard deviations. PI values more than 70% (dotted line) were considered positive for sP antibodies. VN titers greater than 1.65 log (dotted line) were considered positive. Gray circles represent titers for antigens produced in a shaking culture flask, and black circles represent titers for antigens produced in a bioreactor. Statistical comparisons were performed using unpaired Student’s *t*-test (ns = not significant; * *p* < 0.05; ** *p* < 0.01).

The VN titers were <1:45 after the first immunization but increased to >1:45 following the second dose, reaching approximately 1:512 by four weeks post-booster (Figure 5). There was no significant difference in the VN titers between antigens produced in flasks and bioreactors, except for a transient statistical difference three weeks after the second immunization.

## 4. Discussion

FMDV SAT2 was originally endemic to the African continent, but since its emergence in Egypt in 2012, it has spread to Middle Eastern countries and has recently been reported more frequently in Asia [18]. In particular, the spread of SAT2 from Africa to West Asia is likely related to the movement of live animals and livestock products [12]. In the case of the SAT2 outbreak, South Korea stockpiled FMD vaccines overseas in the form of an antigen bank.

In response to the possible incursion of SAT 2 into South Korea, a previous study developed a recombinant SAT2 (SAT2 ZIM-R) vaccine strain and confirmed its protective efficacy in pigs [13]. However, initial experiments focused on evaluating vaccine efficacy using antigens produced in small-scale flasks. To commercialize the SAT2 ZIM-R vaccine strain, we must guarantee that we can produce sufficiently large quantities of vaccine antigens. In this regard, we established optimal conditions for antigen production and scaled-up production using small-scale bioreactors in this study.

To be effective as a vaccine against FMDV, the pentamer, which is composed of five pairs of each of the four FMDV component proteins (VP1, VP2, VP3, and VP4), must assemble into 12 pairs to form 146S particles. Although the component proteins are identical, the protective efficacies of FMDV 146S and 12S (pentamer) particles are known to differ significantly [19]. Therefore, the FMD vaccine antigen measured in this study was 146S, an intact FMDV particle.

Based on previous reports showing that FMDV exhibits increased stability at higher pH levels [18], we adjusted the pH of the culture medium during viral inoculation. The highest antigen productivity was observed at a pH of 7.5. In the case of SAT2 viruses, it has been reported that the thermostability of the virus is highest at a pH of 8.2 [20]. Although FMDVs are generally more stable at a pH >7.0, viral stability and antigen productivity are not always directly correlated, and the optimal conditions for antigen production may differ among viral strains.

Scaling-up the production process in fivefold increments from flask cultures under optimal pH conditions resulted in antigen productivity >7 µg/mL at all scales. Notably, productivity of 5.6 µg/mL was achieved in 1 L cultures using a bioreactor. This productivity is significantly higher than those reported in previous studies, indicating that SAT2 ZIM-R has strong potential for commercial applications. Many studies have reported antigen productivity of approximately 1 µg/mL from viral supernatants [14,21,22], while productivity of about 3 µg/mL has been reported when using bioreactors [23,24]. It has also been reported that increasing the volume of bioreactors does not significantly affect antigen productivity during vaccine antigen production [23,25]. Given that we achieved 5.6 µg/mL in a bioreactor culture, it is expected that higher antigen productivity can be reproduced in large-scale bioreactors for commercialization after a domestic FMD vaccine manufacturing facility is established in the near future.

Since FMD vaccines are produced from large quantities of infectious viruses under BSL-3 containment, viral inactivation kinetics must be performed during the antigen production process, as well as innocuity tests on the finished product [17]. In this study, we assessed the inactivation kinetics by treating the SAT2 ZIM-R virus with different concentrations of BEI at 26 °C and 37 °C [26]. As expected, the virus was more efficiently inactivated at 37 °C than 26 °C, consistent with the previous results [21,22,27,28]. The optimal inactivation condition for SAT2 ZIM-R was identified to be treatment with 1 mM BEI at 26 °C, which is a lower BEI concentration than that (2 mM BEI at 26 °C) previously reported for the FMD type O, A, and Asia1 viral strains [26]. In addition, another study reported that inactivation kinetics of FMDV types O, A, and Asia1 revealed an optimal BEI concentration of 1.2–1.6 mM at 37 °C [21], confirming that the SAT2 ZIM-R in this study was more easily inactivated than other serotypes of FMDV.

In this study, SAT2 ZIM-R exhibited 9% antigen loss with 1 mM BEI at 26 °C, which was the optimal condition for SAT2 ZIM-R inactivation. This is a somewhat greater loss than that identified in our previous study, which revealed antigen losses < 3% with 2 mM BEI treatment at 26 °C for FMDV type O and A (26), indicating that SAT viruses are less thermostable than other serotypes of FMDV [29]. However, this result is better than those in previous reports, with antigen losses > 60% with 1 mM BEI treatment at 37 °C for FMDV types O, A, and Asia 1 in India [27].

Shaking flasks and bioreactors are important tools for bioprocessing but are different in many respects. While bioreactor cultures are stirred for mixing, shaking flasks are agitated using mechanical shakers, usually involving orbital motion. To determine whether viral antigens prepared by these two different methods differ in immunogenicity, we evaluated the immunogenicity of the SAT2 ZIM-R experimental vaccine made from antigens produced in a flask and in a bioreactor. No differences in the VN titers were observed between the antigens derived from flasks and those derived from bioreactors, revealing that production of the FMD vaccine with SAT2 ZIM-R did not compromise vaccine efficacy, regardless of the culture equipment employed.

Following this standard two-dose regimen, neutralizing antibody titers increased up to approximately 500-fold, indicating that the SAT2 ZIM-R vaccine is expected to provide effective immune protection, considering the correlation between neutralizing antibody titers and a vaccine’s protective efficacy [30,31].

In summary, successful large-scale production and commercialization of the SAT2 vaccine strain depends on optimizing several key factors: maximizing antigen productivity, ensuring reliable inactivation of infectious viruses during the production process, and achieving sufficient immunogenicity in the target species. Protective efficacy in pigs has been demonstrated in a previous study [13], and the present study addressed the remaining aspects required for commercialization. In the future, it will be necessary to evaluate antigen productivity and inactivation kinetics using a larger scale of bioreactors.

## 5. Conclusions

To the best of our knowledge, this is the first study to evaluate the antigen productivity, viral inactivation kinetics, and immunogenicity of the FMDV SAT vaccine strain in pigs. Given that most manufacturing processes are proprietary and rarely disclosed, these scientific findings may promote further research in this field and contribute to the manufacturing of high-quality FMD vaccines. Furthermore, the SAT2 ZIM-R candidate used in this study is expected to serve as a strong vaccine candidate for a domestic antigen bank once a Korean FMD vaccine production facility is established in the near future.

## Figures and Tables

**Table 1:** Amount of SAT2 ZIM-R antigen (146S) in virus-infected supernatants after BEI treatment at 26 °C and 37 °C for 6 h and 24 h. Data are shown as means ± standard deviations (*n* = 3). Statistical comparisons were performed using unpaired Student’s *t*-test.

BEI		26 °C				37 °C			
Concentration	0 h	6 h	*p* Value	24 h	*p* Value	6 h	*p* Value	24 h	*p* Value
0.0 mM BEI	4.19 ± 0.38	4.12 ± 0.18	0.9296	4.06 ± 0.22	0.7733	3.87 ± 0.05	0.3810	3.79 ± 0.26	0.2243
0.5 mM BEI	4.19 ± 0.38	4.14 ± 0.07	0.9694	3.68 ± 0.17	0.0428	3.97 ± 0.10	0.6057	3.64 ± 0.69	0.0743
1.0 mM BEI	4.19 ± 0.38	4.11 ± 0.12	0.9082	3.80 ± 0.07	0.1342	3.90 ± 0.16	0.4403	2.68 ± 0.18	<0.0001
2.0 mM BEI	4.19 ± 0.38	3.89 ± 0.16	0.2767	3.75 ± 0.17	0.0813	4.10 ± 0.13	0.9267	1.95 ± 0.14	<0.0001
3.0 mM BEI	4.19 ± 0.38	3.69 ± 0.13	0.0443	3.43 ± 0.28	0.0022	3.84 ± 0.19	0.3078	1.93 ± 0.31	<0.0001

## Data Availability

The original contributions presented in this study are included in the article. Further inquiries can be directed to the corresponding authors.
